# Rhabdomyolysis secondary to COVID-19 infection and vaccination: a review of literature

**DOI:** 10.3389/fmed.2024.1460676

**Published:** 2024-11-20

**Authors:** Mehdi Karimi, Neda Faal Hamedanchi, Kazem Ansari, Reza Nahavandi, Mahsa Mazdak, Fateme Javaherchian, Pooneh Koochaki, Mahsa Asadi Anar, Mahsa Shirforoush Sattari, Mona Mohamaditabar

**Affiliations:** ^1^Faculty of Medicine, Bogomolets National Medical University, Kyiv, Ukraine; ^2^Faculty of Medicine, Islamic Azad University, Tehran Medical Sciences Branch, Tehran, Iran; ^3^Nano-Biotech Foresight Company Biotechnology Campus, Yazd Stem Cells and Regenerative Medicine Institute, Yazd, Iran; ^4^Department of Biochemical and Pharmaceutical Engineering, School of Chemical Engineering, College of Engineering, University of Tehran, Tehran, Iran; ^5^School of Medicine, Isfahan University of Medical Science, Isfahan, Iran; ^6^School of Medicine, Shahid Sadoughi University of Medical Sciences, Yazd, Iran; ^7^Department of Cancer Biology, Lerner Research Institute, Cleveland Clinic, Cleveland, OH, United States; ^8^Student Research Committee, School of Medicine, Shahid Beheshti University of Medical Sciences, Tehran, Iran; ^9^School of Medicine, Islamic Azad University, Sari, Iran; ^10^Student Research Committee, Islamic Azad University, Sari, Iran

**Keywords:** rhabdomyolysis, autoimmunity, COVID-19, coronavirus vaccine, vaccine side effects, vaccine complications

## Abstract

Rhabdomyolysis (RML), characterized by the breakdown of skeletal muscle fibers and the release of muscle contents into the bloodstream, has emerged as a notable complication associated with Coronavirus disease 2019 (COVID-19) infection and vaccination. Studies have reported an increased incidence of RML in individuals with severe COVID-19 infection. However, the exact mechanisms remain unclear and are believed to involve the host’s immune response to the virus. Furthermore, RML has been documented as a rare adverse event following COVID-19 vaccination, particularly with mRNA vaccines. Proposed mechanisms include immune responses triggered by the vaccine and T-cell activation against viral spike proteins. This study aims to review the current literature on the incidence, pathophysiology, clinical presentation, and outcomes of RML secondary to COVID-19 infection and vaccination. We identify common risk factors and mechanisms underlying this condition by analyzing case reports, clinical studies, and pharmacovigilance data. Our findings suggest that while RML is a relatively rare adverse event, it warrants attention due to its potential severity and the widespread prevalence of COVID-19 and its vaccines. This review underscores the need for heightened clinical awareness and further research to optimize management strategies and improve patient outcomes in this context.

## 1 Introduction

The SARS-CoV-2 virus triggered the Coronavirus disease 2019 (COVID-19) pandemic and has significantly impacted societies and global health systems. This disease has devastated the world, leading to several million infections and more than 7 million deaths (1) Initially, it was thought that the respiratory system was most affected by SARS-CoV-2 infection and that the virus only caused respiratory symptoms such as a mild flu-like illness or severe acute respiratory distress syndrome (ARDS), but further investigation revealed that many organs/systems could be affected in the short and long term (2). One of the complications associated with this disease is rhabdomyolysis (RML), which is increasingly recognized as a remarkable phenomenon. Previous research has shown that RML may be a possible consequence of COVID-19 (3, 4).

Numerous case studies have shown that RML is an early or later severe manifestation of COVID-19 (3, 4). Therefore, it is essential to consider the possibility of RML in individuals who have contracted COVID-19 or been vaccinated, as timely detection and intervention are crucial for managing this potentially fatal disease (5, 6). Therefore, the main objective of this study is to provide a comprehensive analysis of the epidemiology, pathophysiology, clinical presentation, laboratory observations, and therapeutic approaches related to RML as a complication of COVID-19 infection.

## 2 Methodology and literature search

A structured approach was followed to gather relevant studies on RML secondary to COVID-19 infection and vaccination for this narrative review. Essential keywords and medical subject headings (MeSH) such as “COVID-19 infection,” “vaccination,” “complications,” and “rhabdomyolysis” were identified to guide the searches through major databases like PubMed and Google Scholar. A wide range of peer-reviewed articles, clinical reports, and case studies were captured. Studies published in English since the onset of the pandemic in 2019 that specifically addressed RML in relation to COVID-19 or its vaccines were included, while papers unrelated to the topic, non-peer-reviewed articles, and those beyond the scope of inquiry were excluded.

After the literature was collected, each study’s design, population, clinical outcomes, and treatment strategies were carefully reviewed. Patterns in the presentation of RML in patients with COVID-19 or following vaccination were identified, along with potential complications. Gaps in the research, such as the limited number of large-scale studies and the need for further investigation into the mechanisms underlying this condition, were noted. A synthesis of these findings was completed to provide an overview of current knowledge and insights into future research needs. This review of literature, using the SANRA (Scale for the Assessment of Narrative Review Articles) scale, resulted in a total score of 9.5/12, reflecting a high standard of quality.

## 3 An overview of rhabdomyolysis

RML is a pathological condition characterized by the breakdown of skeletal muscle tissue and the consequent release of intracellular muscle components, including electrolytes, enzymes, and myoglobin, into the circulatory system, potentially causing systemic complications (1). RML can arise from diverse sources, such as trauma, extended muscle compression, ischemia, drug-induced toxicity, metabolic irregularities, and infections, all of which inflict damage on muscle cell membranes, compromising the structural integrity of muscle fibers (7). A common factor between traumatic and non-traumatic forms of RML is muscle necrosis. RML can have a range of repercussions on the body, from minor elevations in muscle enzymes in the bloodstream without any symptoms to serious, potentially fatal consequences, such as acute renal injury (AKI) and abnormalities in electrolyte balance (8). In individuals with COVID-19, RML has been documented as a potential delayed complication, presenting symptoms such as fatigue, myalgia, and discomfort in the lower extremities. It may be linked to COVID-19 infection and, in rare instances, can occur after COVID-19 vaccination (9, 10). RML is prevalent, with most cases not requiring intensive care unit-level interventions. Rapid recognition and management of RML are crucial to minimizing the risk of serious complications, such as renal failure and electrolyte disturbances (11).

### 3.1 Etiology and Pathophysiology of RML

Numerous factors play a role in the onset of RML, including direct infiltration and toxic breakdown of muscle fibers due to various factors, such as physical trauma, strenuous exercise, medications, drugs, infectious pathogens, metabolic irregularities, electrolyte imbalances (e.g., hypokalemia, hypophosphatemia), genetic anomalies, or as a sequela of viral infections such as SARS-CoV-2 (7, 12). The distinguishing pathophysiological feature of the syndrome involves an elevation in intracellular free ionized calcium levels, stemming from either cellular energy depletion or direct rupture of the plasma membrane. This elevated intracellular calcium level triggers various proteases, enhances the contractility of skeletal muscle cells, leads to mitochondrial dysfunction, and increases the generation of reactive oxygen species, culminating in the death of skeletal muscle cells (13).

#### 3.1.1 Muscle cell injury and intracellular contents release

Following muscle cell injury, intracellular components such as myoglobin, enzymes (lactate dehydrogenase and creatine kinase), electrolytes (phosphate and potassium), and other proteins are released into the bloodstream (14). Myoglobin, a heme-containing protein in muscle cells, plays a significant role in the pathophysiology of RML and is among the primary substances released. Typically, myoglobin exhibits a weak binding affinity with plasma globulin and is excreted in small amounts in urine. However, myoglobin’s excessive release overwhelms the plasma proteins’ binding capacity during RML. Elevated levels of myoglobin in the blood can lead to kidney injury and contribute to the formation of toxic compounds, resulting in metabolic acidosis. Consequently, myoglobin undergoes glomerular filtration and reaches the renal tubules, potentially leading to renal impairment and dysfunction (15).

#### 3.1.2 Changes in cellular metabolism

RML induces significant changes in cellular metabolism, including a surge in creatine kinase (CK) levels due to muscle cell damage; electrolyte imbalances resulting from the release of potassium, phosphate, and myoglobin into the bloodstream; and metabolic acidosis triggered by toxic compounds formed by myoglobin release. Additionally, myoglobin release can cause acute kidney injury by damaging renal tubules, whereas elevated potassium levels from muscle cell damage can lead to hyperkalemia, potentially causing cardiac arrhythmias (16). Muscle injury initiates a series of physiological processes that release extracellular calcium ions into the intracellular compartment. Excessive amounts of calcium ions in the body may result in abnormal interactions between contractile proteins, myosin, and actin. This, in turn, can contribute to the degradation and damage of muscle fibers and the overall muscle tissue (17). In typical myocytes, Na-K-ATP-ase pumps are inside the cellular membrane, facilitating the active transportation of sodium ions to the extracellular space. This process contributes to establishing a negative membrane potential (18). Additionally, it should be noted that the Na+/Ca2+ exchanger is located inside the cellular membrane. The pump facilitates the re-entry of sodium ions into the cell while concurrently transporting calcium ions out of the cell (19). This mechanism depends on adenosine triphosphate (ATP) as a primary energy source. The central mechanism underlying RML is the influx of calcium into the cell, which is triggered by ATP depletion and cell membrane breakdown. The activation of phospholipase A2, proteases, and vasoactive molecules by excess intracellular calcium leads to the generation of free radicals (20).

### 3.2 Reperfusion injury

Most injuries tend to develop after blood flow is restored to a specific location of the damage. Reperfusion outcomes include the transportation of activated neutrophils to tissues previously deprived of blood flow, leading to the generation of free radicals originating from reactive oxygen species. These free radicals can potentially harm the cell membrane’s lipid bilayer via lipid peroxidation (21).

### 3.3 Clinical presentation of rhabdomyolysis

The traditional clinical triad associated with RML comprises muscular weakness, myalgia, and black urine (14). However, it should be noted that not all patients exhibit these symptoms (22). Numerous clinical manifestations of RML exhibit a lack of specificity and encompass a spectrum of presentations, ranging from asymptomatic cases with elevated enzyme levels to critical conditions characterized by multiple electrolyte imbalances such as hyperphosphatemia, hyperkalemia, hypocalcemia, severe depletion of intravascular volume, metabolic acidosis, and acute renal failure. The clinical presentation of classical triads includes manifestations such as myalgia, general discomfort, and the presence of urine with a characteristic tea-like coloration. Nevertheless, it has been documented that these symptoms occur in less than 10% of individuals (17, 23, 24).

### 3.4 Diagnosis of rhabdomyolysis

The identification of RML necessitates clinical suspicion, and clinicians need to consider this diagnosis consistently, particularly in patients exhibiting a typical trio with established risk factors. Timely identification of this ailment contributes to the mitigation of associated problems (16). Measurement of serum creatine kinase (CK) level is often regarded as the most reliable and widely accepted laboratory diagnostic test (11). Serum CK levels are often below 100 U/L. No established threshold exists for creatine kinase levels; however, readings >1,000 U/L indicate RML. Many doctors choose values between three and five times the upper normal range, which is between 100 and 400 IU/L (or approximately 1,000 IU/L) (1). Elevated serum CK levels have been identified as predictive factors for the development of acute renal failure (25). However, using myoglobin as a conventional diagnostic test is limited by its short half-life. However, an elevated concentration of myoglobin in individuals suspected of having RML may indicate the first stage of the condition (24, 26). Additional diagnostic methods for RML include myoglobinuria and positive urine dipstick tests with an orthotolidine (OT) test (27).

### 3.5 Management of RML

Addressing the root cause of muscle injury is the initial step in managing RML. The primary approach to treating RML involves providing supportive care, primarily by ensuring sufficient hydration to prevent acute renal failure. Early and vigorous administration of fluid replacement with crystalloid solutions is pivotal for controlling and managing AKI resulting from RML. Electrolyte imbalance should be managed using standard medical protocols (1). Intravenous fluid resuscitation can help prevent renal problems. Hypovolemia can worsen acute renal failure. Hemodynamic monitoring is essential for cardiovascular or renal illness (28). Administering 0.9% sodium chloride solution before extricating entrapped individuals can reduce the occurrence of acute renal failure. Alkalizing urine can prevent myoglobin deposits from forming, which can lead to renal failure (29). Diuretics like mannitol can reduce muscle swelling and hypovolemia. Traditional medicines like bicarbonate therapy and dextrose-insulin are used for managing hyperkalemia (30). Hemodialysis is effective for managing electrolyte imbalances. Hemodiafiltration and continuous renal replacement approach help manage cardiovascular instabilities in patients (29).

### 3.6 Complications of RML

The significance of complications associated with RML stems from the fact that the majority of clinical manifestations seen in cases of RML may be attributed to these complications. Complications can be categorized into two distinct classes: early and late complications (31). Early consequences include hyperkalemia, hypocalcemia, hepatic inflammation, cardiac arrhythmia, and cardiac arrest, as stated in the literature (17). Late concerns, on the other hand, include acute renal failure and disseminated intravascular coagulation. The spectrum of complications varies from slight increases in creatinine phosphokinase levels to severe medical crises such as compartment syndrome, intravascular fluid depletion, disseminated intravascular coagulation, pigment-induced acute kidney injury (AKI), and cardiac arrhythmias (1, 32).

## 4 An overview of COVID-19 infection

COVID-19 is an infectious respiratory illness caused by the novel SARS-CoV-2. It was first identified in December 2019 in Wuhan, China, and has since spread globally, leading to a pandemic (33). The SARS-CoV-2 virus primarily spreads through respiratory droplets when an infected person coughs, sneezes, or talks. It can also spread by touching surfaces contaminated with the virus and touching one’s face, particularly the mouth, nose, or eyes (34). Several factors increase the risk of contracting COVID-19, including close contact with infected individuals, especially in enclosed spaces with poor ventilation. Other risk factors include advanced age, underlying health conditions such as cardiovascular disease, diabetes, obesity, and compromised immune systems (35). COVID-19 can lead to various complications, ranging from mild to severe. Common complications include pneumonia, ARDS, multi-organ failure, sepsis, blood clots, and neurological complications. Specific individuals, particularly older adults and those with underlying health conditions, are at higher risk of developing severe complications (36, 37).

### 4.1 Diagnosis and management of COVID-19 infection

Diagnosis of COVID-19 typically involves a combination of clinical symptoms, laboratory testing, and imaging studies. Common symptoms include fever, cough, shortness of breath, fatigue, muscle aches, loss of taste or smell, sore throat, and headache (38). Laboratory tests, such as polymerase chain reaction (PCR) or antigen tests, are used to detect the presence of the SARS-CoV-2 virus in respiratory samples (39, 40). Chest X-rays or computed tomography (CT) scans may be performed to assess lung involvement in severe cases (41). Treatment for COVID-19 varies depending on the severity of the illness. Mild cases may only require supportive care, such as rest, hydration, and over-the-counter medications to relieve symptoms. In moderate to severe cases, hospitalized patients may receive oxygen therapy, corticosteroids to reduce inflammation, antiviral medications like remdesivir, and other supportive treatments. Intensive care measures such as mechanical ventilation and extracorporeal membrane oxygenation (ECMO) may be necessary in critically ill patients (41–43).

### 4.2 COVID-19 vaccination

The COVID-19 vaccination stands as a crucial tool in the global endeavor to combat the ongoing pandemic. Different COVID-19 vaccines employ diverse technologies to elicit immune responses against the SARS-CoV-2 virus (44). Different types of COVID-19 vaccines are available, including mRNA, viral vector, protein subunit, and inactivated or attenuated virus vaccines. All vaccines aim to trigger immunity against the virus and require one or two doses. Vaccination is crucial in achieving widespread immunity and controlling the spread of the disease (45, 46). The primary technologies behind COVID-19 vaccines include mRNA-based vaccines, such as those developed by Pfizer-BioNTech and Moderna, and viral vector vaccines, like the AstraZeneca and Johnson & Johnson vaccines (47). Additionally, studies have shown that COVID-19 vaccines can lead to regional immune reactions, as evidenced by changes in 18F-FDG PET/CT scans, which can help understand the vaccines’ immunogenicity (47).

## 5 Association between COVID-19 infection and rhabdomyolysis

COVID-19 has been associated with increased RML, characterized by the breakdown of skeletal muscle tissue and the release of muscle cell content into the bloodstream. RML is a significant complication associated with COVID-19 since it has the potential to exacerbate patients’ clinical manifestations and may result in mortality (24). It has been identified as an initial clinical presentation and subsequent outcome of SARS-CoV-2 infection (4). Several studies have documented instances of RML in COVID-19 patients, particularly in those with severe illness or complications, underscoring the importance of recognizing this potentially life-threatening manifestation of the disease (4, 48–50). The precise relationship between COVID-19 and RML remains incompletely understood; however, it is believed to be linked to the body’s immune response to the virus.

A retrospective analysis of 1,079 COVID-19 patients admitted to the ICU investigated their features and outcomes. The study discovered that acute kidney damage (AKI) was the most important predictor of mortality among patients infected with COVID-19 and undergoing rhabdomyolysis. RML was linked to a higher risk of death in ICU patients with COVID-19, with acute renal injury being the best predictor of fatal outcomes. These findings emphasize the vital need for early detection and treatment of RML in individuals with severe COVID-19 (51).

Table 1 presents the summary of variables and outcomes of case report studies of RML secondary to COVID-19 infection and vaccination (5, 6, 9, 48–50, 52–107). Table 2 summarizes cases of RML secondary to COVID-19 infection and vaccination and its associated outcomes.

**Table 1:** Variables and outcomes of patients with rhabdomyolysis secondary to COVID-19 infection and vaccination.

Variables	Measurements
Age (year), Median (IQR) (*n* = 76)	47 (32.5–65.5)
Male (%) (*n* = 76)	60 (78.9%)
Past myotoxic DH, Positive (%) (*n* = 33)	18 (54.5%)
Peak CK (IU/L), Median (IQR) (*n* = 76)	22,000 (7195.5–81,030)
AST (IU/L), Median (IQR) (*n* = 36)	407.5 (180.75–1744.75)
ALT (IU/L), Median (IQR) (*n* = 29)	216 (88.5–441)
LDH (IU/L), Median (IQR) (*n* = 39)	981 (550–2,750)
Peak Creatinine (mg/Dl) Median (IQR) (*n* = 62)	2.84 (1.17–11.39)
Hematuria (*n* = 34)	29 (85.2%)
Outcome (Expired) (%) (*n* = 76)	19 (25%)

CK, creatine kinase; DH, drug history; AST, Aspartate Aminotransferase; ALT, Alanine Aminotransferase; LDH, Lactate Dehydrogenase.

**Table 2:** Cases of rhabdomyolysis secondary to COVID-19 infection and vaccination.

Author/year	Description	Ref.
Alrubaye and Choudhary (2020)	A 35-year-old female with no significant past medical history presented with myalgia, fever, chills, cough, dyspnea, diarrhea, and dark urine. Lab results revealed a peak CK of 71,000, AST/ALT of 1900/450, LDH of 401, and creatinine of 0.82. The patient survived the episode.	(52)
Anklesaria et al. (2020)	A 57-year-old patient with a history of obesity class 2, HTN, and HLP presented with fever, malaise, and myalgia. The patient was on Hydralazine 50 (BID), Furosemide 40 (QD), and Rosuvastatin 5 (QD) with positive past myotoxic DH. Lab results showed a peak CK of 1,083,744, AST/ALT of 3995/751, and creatinine of 7.6 with positive blood test in U/A. The patient expired.	(53)
Chan et al. (2020)	A 75-year-old female with CAD, HTN, and GERD presented with malaise, myalgia, and anorexia. Lab results revealed a peak CK of 2,767, AST/ALT of 198/63, LDH of 497, and creatinine of 1.2 with positive blood test in U/A. She survived the episode.	(54)
Chan et al. (2020)	A 71-year-old male with hypertension, seizure disorder, CKD, and overactive bladder presented with generalized weakness and leg twitching. Lab results showed a peak CK of 1859, LDH of 538, and creatinine of 3.6 with positive blood test in U/A. The patient had an unknown outcome.	(54)
Chedid et al. (2020)	A 51-year-old male with HTN, T2DM, obstructive sleep apnea, and CKD stage-2 presented with diffuse myalgia, dry cough, and mild chills. Lab results revealed a peak CK of 464,000, AST/ALT of 715/122, LDH of >2,150, and creatinine of 2.48 with positive blood test in U/A. The patient survived.	(55)
Suwanwongse and Shabarek (2020)	An 88-year-old male with HTN, CKD, HFrEF, BPH, and mild cognitive impairment presented with acute pain and weakness in the lower extremities, fever, and dry cough. The patient was on Simvastatin, Donepezil, Furosemide, Losartan, Metoprolol Succinate, Tamsulosin with positive past myotoxic DH. Lab results revealed a peak CK of 13,581, LDH of 364, and creatinine of 1.38 with positive blood test in U/A. The patient survived.	(56)
Jin and Tong (2020)	A 60-year-old male presented with fever, cough, and weakness in the lower extremities. Lab results revealed a peak CK of 17,434, AST/ALT of 373/172, LDH of 2,347, and creatinine of 74.4 with positive blood test in U/A. The patient survived.	(9)
Mukherjee et al. (2020)	A 49-year-old male with T2DM and HTN presented with fever, chills, dyspnea, myalgia, cough, hyponatremia, and hypokalemia. Lab results revealed a peak CK of 23,060, AST/ALT of 160/470, LDH of 955, and creatinine of 1.18 with positive blood test in U/A. The patient survived.	(57)
Anwar and Al Lawati (2020)	A 16-year-old male with a history of viral myositis and rhabdomyolysis presented with fever, sore throat, myalgia, and dyspnea. The patient expired.	(58)
Arai, Murata et al. (2021)	A 61-year-old male with hypertension presented with hypotension and a constant high fever. Lab results revealed a peak CK of 8,318. The patient survived.	(59)
Aria et al. (2020)	A 50-year-old male with hypothyroidism and migraine presented with severe progressive headache, nausea, and vomiting. Lab results revealed a peak CK of 41,240, AST/ALT of 850/144, and creatinine of 3.3 with positive blood test in U/A. The patient expired.	(60)
Uysal et al. (2020)	A 60-year-old male presented with myalgia and fatigue. Lab results revealed a peak CK of 4,267, AST/ALT of 137/208, LDH of 781, and creatinine of 0.9. The patient survived.	(61)
Byler et al. (2021)	A 67-year-old female with HTN, T2DM, and HLP presented with chest heaviness, cough, worsening dyspnea, fatigue, chills, and fever. The patient was on statin with positive past myotoxic DH. Lab results revealed a peak CK of 15,085, AST/ALT of 749/337, and creatinine of 10.95 with positive blood test in U/A. The patient survived.	(6)
Cassim et al. (2021)	A 12-year-old female presented with fever, sore throat, myalgia, mild dyspnea, and inability to walk. Lab results revealed a peak CK of >22,000, AST/ALT of 2227/457, and LDH of 614 with positive blood test in U/A. The patient survived.	(62)
Rivas-García et al. (2020)	A 78-year-old male with T2DM and HTN presented with asthenia, fever, severe myalgia, muscle weakness, and dark-colored urine. Lab results revealed a peak CK of 22,511, AST/ALT of 972, and creatinine of 3.2 with positive blood test in U/A. The patient survived.	(63)
Bach et al. (2021)	A male case with Idiopathic biphasic anaphylaxis with rhabdomyolysis (6 months ago) presented with headache, myalgia, anosmia, anorexia, dyspnea and wheezing, urticaria, and edema. Lab results revealed a peak CK of 174,300, and creatinine of 0.8 with positive blood test in U/A. The patient survived.	(64)
Azeem et al. (2021)	A 67-year-old male with T2DM, HTN, and gout presented with severe weakness and loss of consciousness. Lab results revealed a peak CK of 40,000 and creatinine of 5.45. The patient expired.	(65)
Fadel et al. (2021)	A 33-year-old male presented with dark-colored urine, myalgia, intractable headache, ageusia, and dyspnea. Lab results revealed a peak CK of 362,445, AST/ALT of 1835/190, LDH of >4,000, and creatinine of 1 with a positive blood test in U/A. The patient survived.	(66)
Fadiran (2021)	A 78-year-old female with depression, bipolar disorder, mild cognitive impairment, hypothyroidism, and dyslipidemia presented with profound weakness, hypotension, and fever. Lab results revealed a peak CK of 10,650 and LDH of 550. The patient survived.	(67)
Cunha et al. (2020)	A 46-year-old female with breast cancer presented with fever, nausea, vomiting, abdominal pain, diarrhea, dry cough, asthenia, and myalgia. The patient was on Neoadjuvant chemotherapy (Paclitaxel) with positive past myotoxic DH. Lab results revealed a peak CK of 87,456, AST/ALT of 442/216, and LDH of 2,750. The patient survived.	(68)
Egoryan et al. (2021)	A 50-year-old male with T2DM, HTN, hyperlipidemia, legal blindness, and deafness presented with constipation, oliguria, and dark-colored urine.The patient was on Atorvastatin 20 mg (QD), Metformin 500 mg (QD), Lisinopril 5 mg (QD) with positive past myotoxic DH. Lab results revealed a peak CK of 359,910, AST/ALT of 2222/432, LDH of >6,000, and creatinine of 3.51 with positive blood test in U/A. The patient survived.	(69)
Chiba et al. (2021)	A 36-year-old male presented with cough, fever, and worsening dyspnea. Lab results revealed a peak CK of 26,046, LDH of 220, and creatinine of 0.7. The patient survived.	(70)
Khosla et al. (2020)	A 65-year-old male with HTN, HLP, and obstructive sleep apnea presented with dyspnea, cough, and diarrhea. The patient was on Lisinoprol pravastatin 40 mg with positive past myotoxic DH. Lab results revealed a peak CK of 7,854 creatinine of 5.3. The patient expired.	(71)
Khosla et al. (2020)	A 78-year-old male with T2DM, HTN, HLP, and CAD presented with somnolence, hypoxia, lactic acidosis, hyperkalemia, and hypotension. The patient was on Atorvastatin 40 mg, Metformin with positive past myotoxic DH. Lab results revealed a peak CK of >22,000, LDH of >2,500 and creatinine of 1.4. The patient expired.	(71)
Khosla et al. (2020)	A 67-year-old male with T2DM, HTN, HLP, and CKD stage-4 presented with altered mental status, hypotension, and hypoxia. The patient was on Atorvastatin 40 mg with positive past myotoxic DH. Lab results revealed a peak CK of 6,164, LDH of 624, and creatinine of 24.3. The patient survived.	(71)
Khosla et al. (2020)	A 58-year-old male with T2DM, HTN, and HLP presented with headaches, worsening dyspnea, and dry cough. The patient was on Atorvastatin 20 mg, Metformin with positive past myotoxic DH. Lab results revealed a peak CK of 4,625, LDH of 845 and creatinine of 24.3. The patient survived.	(71)
Khosla et al. (2020)	A 64-year-old male with T2DM, HTN, and HIV presented with dyspnea, nausea, vomiting, fever, and hypoxia. The patient was on Metformin, Odefsey with neagtive past myotoxic DH. Lab results revealed a peak CK of 3,135, LDH of 384, and creatinine of 1. The patient survived.	(71)
Wu et al. (2021)	A 74-year-old female with TDM2 and HTN presented with fever. Lab results revealed a peak CK of 1,663 and creatinine of 1.3 with a positive blood test in U/A. The patient survived.	(72)
Fabi et al. (2021)	A 6-year-old female presented with tenderness, abdominal pain, diarrhea, chest pain, severe myalgia, and abnormal gait. Lab results revealed a peak CK of 3,392 and creatinine of 1.11. The patient survived.	(73)
Singh-Kucukarslan and Heidemann (2020)	A 42-year-old patient presented with subacute lower back pain and leg pain. Lab results revealed a peak CK of 313,700, AST/ALT of 1424/273, LDH of 14,007 and creatinine of 9.8 with positive blood test in U/A. The patient survived.	(74)
Foster et al. (2020)	A 40-year-old male with HIV, HTN, and obesity presented with hypotension, dyspnea, fatigue, leg cramping, poor appetite, fever, chills, and cough. The patient was on Highly active antiretroviral therapy (HAART) with positive past myotoxic DH. Lab results revealed a peak CK of 3,127 and creatinine of 17.5 with positive blood test in U/A. The patient survived.	(75)
Valente-Acosta et al. (2020)	A 71-year-old male with BPH presented with dry cough, mild dyspnea, fever, severe myalgia, arthralgia, generalized weakness, and malaise. Lab results revealed a peak CK of 8,720, LDH of 672 and creatinine of 1.68 with positive blood in U/A. The patient survived the episode.	(76)
Chong and Saha (2020)	A 37-year-old male presented with dyspnea and fatigue. Lab results showed an elevated CK level of 35,000, AST/ALT levels of 7,600, and a creatinine level of 5. The patient expired.	(77)
Samies et al. (2020)	A 16-year-old male with a past medical history of obesity, hypertension, T2DM, and obstructive sleep apnea presented with intermittent fever, sore throat, nonproductive cough, myalgia, and dark-colored urine. The patient was on Lisinopril, Hydrochlorothiazide, Metformin with positive past myotoxic DH. The patient had a CK level of 426,700 and creatinine of 12.03. He survived the episode.	(78)
Mahmood et al. (2021)	An 81-year-old female with a history of hypertension, T2DM, hyperlipidemia, and CKD stage III, on Atorvastatin 40 mg, Losartan, Glipizide, and Metformin, presented with malaise, myalgia, anorexia, dry cough, and mild dyspnea. Lab results showed a CK of 40,900 and a positive urine test for blood. She survived the episode.	(79)
Legrand et al. (2020)	A 39-year-old male presented with chest pain and dyspnea. Lab results showed elevated CK of 17,070 and AST/ALT levels of 556/557. Creatinine was at 113. He survived the episode.	(80)
Mah et al. (2021)	A 32-year-old male with bilateral L5 nerve root radiculopathy presented with dry cough, rhinorrhea, fever, bilateral upper chest pain, and bilateral leg pain. Lab results revealed a peak CK of 11,071, LDH of 1,032, and creatinine of 66. He survived the episode.	(81)
Meegada et al. (2020)	A 19-year-old male with ulcerative colitis, non-adherent to prescribed mesalamine, presented with diffuse abdominal pain, particularly in the left upper quadrant, headache, and severe back pain. The patient was non-adherence to the prescribed mesalamine with negative past myotoxic DH. Lab results showed a CK of 22,000 and creatinine of 1.28. He survived the episode.	(82)
Jahnke et al. (2021)	A 69-year-old female with a history of hypertension, acute MI, Alzheimer’s disease, and cardiopathy presented with dyspnea and diarrhea. Lab results showed a CK level of 5,014 and creatinine of 4.8 with a positive urine test for blood. She expired.	(83)
Li et al. (2021)	A 22-year-old female with a history of asthma presented with a sore throat, myalgia, and 24 h of anuria. Lab results showed a CK level of 760,000 and a positive urine test for blood. She survived the episode.	(84)
Murillo et al. (2020)	A 48-year-old male presented with malaise, myalgias, asthenia, dry cough, progressive dyspnea, and fever. His CK was elevated at 10,768. The outcome is unknown.	(85)
Patel et al. (2021)	A 60-year-old male with a history of obesity, CKD stage II, and asthma presented with malaise, myalgia, anorexia, and a mild nonproductive cough. The patient was a Mometasone-Formoterol inhaler, Cyclobenzaprine, and Sildenafil with negative past myotoxic DH. His CK level was 685,000, and his creatinine was 5.88. He expired.	(86)
Su and Kamangar (2020)	A 55-year-old male with a history of hypertension and hyperlipidemia presented with dyspnea, dry cough, and fevers. His CK was elevated at 12,391. He survived the episode.	(87)
Su and Kamangar (2020)	A 59-year-old male with stage 0 chronic lymphocytic leukemia, hyperlipidemia, and GERD, who had been intubated for influenza B, presented with malaise and myalgia. His CK was elevated at 4617 with a positive blood test in U/A. He survived the episode.	(87)
Taxbro et al. (2020)	A 38-year-old male with T2DM, gout, and obesity presented with fever, myalgia, nausea, vomiting, dry cough, dyspnea, and abdominal pain. He survived the episode.	(88)
Tram et al. (2020)	A 15-year-old male presented with malaise, myalgia, abdominal pain, vomiting, mild diarrhea, hematuria, polyuria, and polydipsia. His CK level was 21,876, AST/ALT levels of 319/118 and creatinine was 8.91. He survived the episode.	(89)
Fujita et al. (2021)	A 19-year-old female presented with a dry cough, high fever, myalgia, and malaise. Lab results showed elevated CK of 55,613, AST/ALT of 1013/252, LDH of 1,583, and creatinine of 0.7. She survived the episode.	(90)
Gefen et al. (2020)	A 16-year-old male with autism, hyperactivity disorder, morbid obesity, and obstructive sleep apnea presented with fever, myalgias, dyspnea with exertion, and dark-colored urine. His CK was 427,656, with a positive blood test in U/A. He survived the episode.	(49)
Gilpin et al. (2021)	A 16-year-old male with a history of asthma presented with bilateral pain in the shoulders and thighs, and dark-colored urine. His CK level was 392,488, AST/ALT of 2055/426, LDH of 13,942 with positive blood test in U/A. He survived the episode.	(91)
Heidarpour et al. (2021)	A 22-year-old male presented with spastic muscles in the extremities. Lab results showed elevated CK of 1,840, AST/ALT of 261/110, LDH of 1740 and creatinine of 1.2. He expired.	(92)
Heidarpour et al. (2021)	A 36-year-old male with psychosis, on Olanzapine, Lorazepam, Propranolol, and Sertraline, presented with polyuria, polydipsia, nausea, and vomiting. His CK level was 5,130 and LDH was 1,296. He survived the episode.	(92)
Heidarpour et al. (2021)	A 47-year-old female with DM, hypertension, hypothyroidism, and asthma, presented with fever, cough, and dyspnea. Lab results showed CK of 16,480, AST/ALT of 951/874, LDH of 5,580 and creatinine of 1.7. She expired.	(92)
Husain et al. (2020)	A 38-year-old male presented with fever, cough, dyspnea, and myalgia. His CK was 33,000, AST/ALT of 61/58, LDH of 398 and creatinine was 1.5. The outcome is unknown.	(48)
Zhang et al. (2020)	A 38-year-old male with GERD and sleep apnea presented with dyspnea and fever. His CK level was >42,670. He survived the episode.	(93)
Flato et al. (2021)	An 18-year-old female with grade 3 obesity presented with dry cough, dyspnea, fever, and myalgia of the lower limbs. Her CK level was 23,735, AST/ALT of 208/NA, LDH of 1,080 and creatinine was 6.01. She expired.	(94)
Kartalova et al. (2021)	A 73-year-old male with T2DM, essential hypertension, and PCI/stent, on Atorvastatin 20 mg, presented with dry cough, fever, diarrhea, malaise, and severe myalgia, predominantly in the legs. His CK was 6,976, AST/ALT of 300/328, LDH of 981 and creatinine was 96 with positive blood test in U/A. He survived the episode.	(5)
Taneska^1^ et al. (2020)	A 68-year-old male with T2DM, obesity, and acute myocardial infarction is on Metformin, Insulin, Clopidogrel, Spironolactone, Furosemide, Lisinopril, Carvedilol, Rosuvastatin, presented with malaise, myalgia, chest pain, dry cough, dyspnea, and dark-colored urine. His CK level was 1,002, LDH of 559, and creatinine was 689, and he survived the episode.	(95)
Farouji et al. (2021)	A 21-year-old male presented with fever, cough, and dyspnea. Lab results showed CK of 53,886 and creatinine of 0.9. He survived the episode.	(96)
Rosato et al. (2020)	A 58-year-old male with hypertension presented with fever, cough, and dyspnea. Lab results showed a CK of 3,309 and creatinine of 310.35. He survived the episode.	(97)
Sathappan et al. (2020)	A 55-year-old female with a history of traumatic falls in childhood complicated by left hemiparesis, cognitive impairment, DM, DVT, unspecified seizure disorder, and bipolar disorder, on Insulin, Anticoagulants, Antiepileptics, and Antipsychotics presented 10 days after discharge from the hospital for severe hyperglycemia and an acute change in physical mobility. Her CK level was 43,720, and her creatinine of 0.63. She survived the episode.	(98)
Shanbhag et al. (2020)	A 19-year-old male with anxiety and influenza-associated rhabdomyolysis presented with mild dry cough, worsening lower extremities myalgias, and hematuria. Lab results showed a CK level of 694,200, AST/ALT of 2715/483, LDH of 13,950, and creatinine of 0.982. He survived the episode.	(99)
Singh et al. (2020)	A 67-year-old male with hypertension presented with fever and dyspnea. His CK level was 19,773, AST/ALT of 46/30, LDH of 459 and creatinine was 1.16. He expired.	(100)
Singh et al. (2020)	A 37-year-old male with hypertension, on Amlodipine, Clonidine, Hydrochlorothiazide, and Furosemide, presented with fever, myalgia, dyspnea, and altered mental status. His CK level was 4,330, AST/ALT of 131/65, LDH of 907, and creatinine was 3.8 with a positive blood test in U/A. He expired.	(100)
Singh et al. (2020)	A 43-year-old male with end-stage renal disease, on Hydralazine, presented with fever, myalgia, cough, and dyspnea. His CK was 9,793, AST/ALT of 142/43, LDH of 478 and creatinine was 20. He expired.	(100)
Singh et al. (2020)	A 70-year-old male presented with cough and dyspnea. Lab results showed elevated CK of 5,008, AST/ALT of 179/53, LDH of 1,475, and creatinine was 1.68 with a positive blood test in U/A. He expired.	(100)
Solís et al. (2020)	A 46-year-old male with chronic myeloid leukemia, on Imatinib, presented with cough, dyspnea, fever, malaise, and myalgia. His CK was 426,700, AST/ALT of 3565/1432, LDH of 26,867 and creatinine was 16.25. He expired.	(50)
Chetram et al. (2021)	A 62-year-old male with hypertension, morbid obesity (BMI of 39.6), and T2DM, on Aspirin, Glargine, Hydrochlorothiazide-Lisinopril, Pioglitazone, and Simvastatin, presented with malaise, poor appetite, oliguria, and hematuria. His CK level was 327,629, with a positive blood test in U/A. He survived the episode.	(101)
Mughal et al. (2021)	A 39-year-old male presented with ascending bilateral lower limb muscle weakness and severe malaise. His CK level was 36,064. He survived the episode.	(102)
Kontou et al. (2022)	A 10-year-old female presented with pain in the lower extremities and limping. Her CK level was 13,147, AST/ALT of 288/67, LDH of 631. She survived the episode.	(103)
Yu et al. (2022)	A 19-year-old male with obesity, obstructive sleep apnea, and asthma who vaped marijuana presented with malaise, myalgias, decreased appetite, diaphoresis, and dark urine. His CK was 346,695, with a positive blood test in U/A. He survived the episode.	(104)
Donati et al. (2022)	A 38-year-old male presented with dyspnea, lower back pain, lower limb hypoesthesia, and pharyngodynia. Lab results showed CK of 64,682 and creatinine of 11.18. He survived the episode.	(105)
Buckholz et al. (2020)	A 43-year-old male presented with myalgia, cough, and fever. Lab results showed CK of 75,240, AST/ALT of 1,474/NM, and creatinine of 13.35. He survived the episode.	(106)
Buckholz et al. (2020)	A 37-year-old male presented with myalgia, cough, dyspnea, and fever. Lab results showed CK of 82,960, AST/ALT of 902/NM, and creatinine of 1.47. He survived the episode.	(106)
Buckholz et al. (2020)	A 75-year-old male with DVT presented with rhinorrhea, back pain, and weakness. His CK was 3,638, AST/ALT of 56/NM, and creatinine was 1.78. He survived the episode.	(106)
Buckholz et al. (2020)	A 59-year-old male presented with cough, fever, and diarrhea. His CK level was 8,310, AST/ALT of 186/NM, and creatinine was 1.29. He expired.	(106)
Buckholz et al. (2020)	A 66-year-old male with hypertension presented with cough, dyspnea, and fever. His CK level was 10,100, AST/ALT of 263/NM, and creatinine of 1.22. He survived the episode.	(106)
Buckholz et al. (2020)	A patient with multiple myeloma and CKD presented with cough, dyspnea, and malaise. His CK was 406,300, AST/ALT of >6,000/NM, and creatinine was 12.3. He expired.	(106)
Philippe et al. (2022)	A 19-year-old male with major depressive disorder and PTSD, on Mirtazapine, presented with hematuria and dysuria. His CK level was 510,000, AST/ALT of 79/283, and creatinine was 0.92. He survived the episode.	(107)

PMH, Past medical history; DH, Drug history; CK, Creatine Kinase; CKD, Chronic kidney disease stage; T2DM, Type-2 diabetes mellitus; HTN, hypertension; MI, Myocardial infarction; HFrEF, Heart failure with reduced ejection fraction; CAD, Coronary artery disease; PCI, Percutaneous Coronary Intervention; BPH, Benign Prostatic Hyperplasia; HIV, Human immunodeficiency virus; HLP, Hyperkeratosis lenticularis perstans; DVT, Deep vein thrombosis; PTSD, Post-traumatic stress disorder; BMI, Body-Mass-Index.

### 5.1 Mechanism of RML secondary to COVID-19 infection

**Figure 1:**
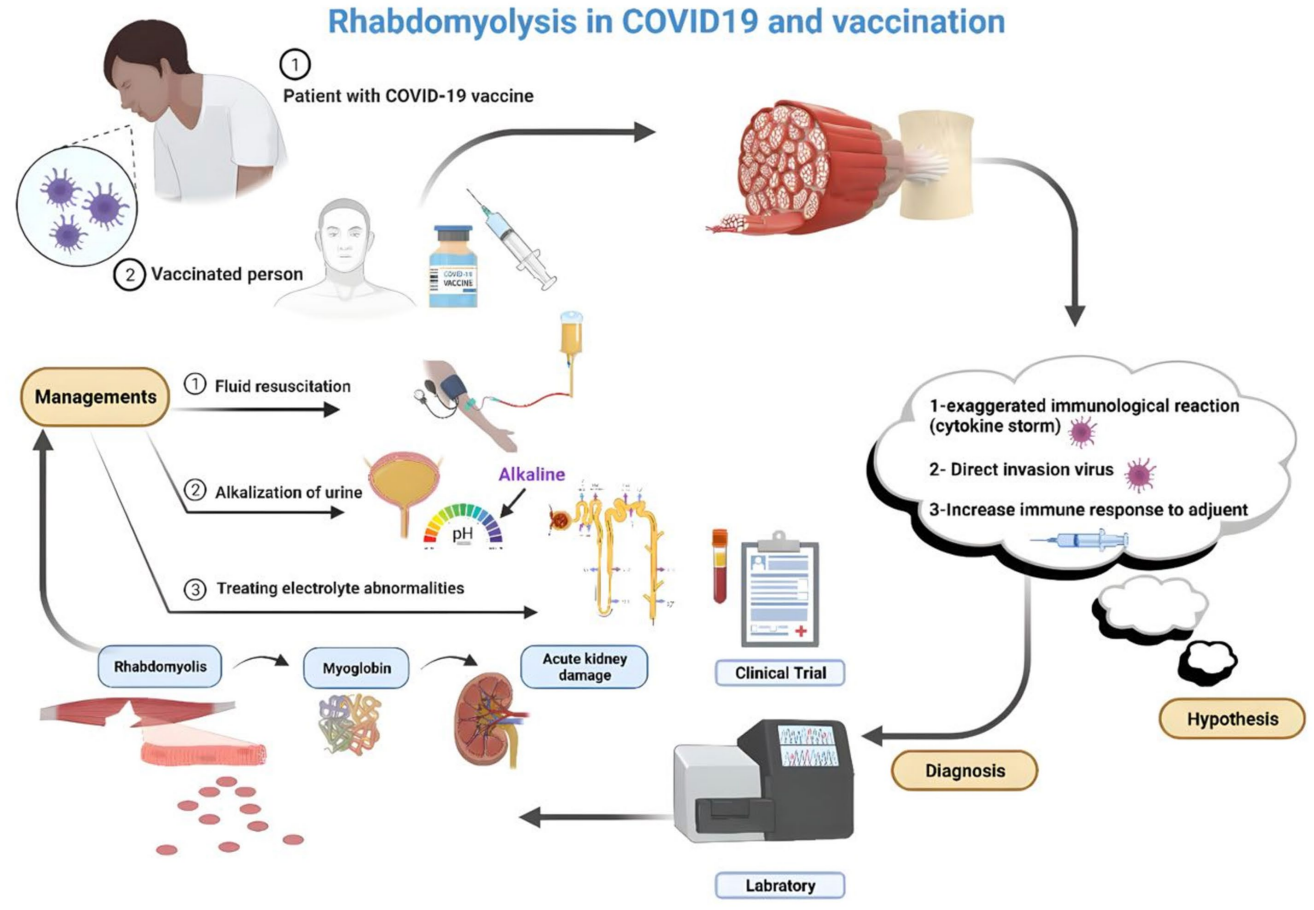
RML hypothesized the pathophysiology after vaccination for COVID-19 (Created by the author using BioRender).

The precise mechanism linking COVID-19 infection to RML remains incompletely understood; however, it is hypothesized to encompass multiple factors, including direct infiltration of muscle cells by the virus, systemic inflammation, cytokine release syndrome, and administration of specific medications in COVID-19 management. One key aspect is the direct invasion of muscles by SARS-CoV-2, causing muscle damage and necrosis and releasing intracellular contents into the bloodstream (90). Nonetheless, two mechanisms have been delineated as complications of viral infections culminating in RML. First, the virus directly invades the muscle cells, leading to their death. Second, cytokines and immunological factors affect the host response, producing toxic effects on muscle cells (88). The mechanisms underlying virus-induced muscle degradation may involve direct viral toxicity and cytokine-mediated muscle impairment (108). Furthermore, in COVID-19 patients, immunological pathways and mechanisms may contribute to viral myositis and subsequent development of RML (82) (see Figure 1).

### 5.2 Clinical presentations of RML secondary to COVID-19 infection

RML is a notable clinical manifestation in individuals with COVID-19, manifesting either as an initial symptom or emerging at any juncture during the illness (56). The clinical manifestations of RML among COVID-19 patients can exhibit considerable variation, encompassing symptoms such as muscular discomfort, weakness, and tenderness, notably concentrated in affected muscle groups (109). Darkened urine attributable to the presence of myoglobin stemming from muscle breakdown may be observed in some instances. Additionally, individuals may report fatigue, general malaise, and challenges in mobility or executing routine activities (110). In certain instances, the discovery of RML among COVID-19 patients may be serendipitous, alongside predominant symptoms, such as cough and fever. Vigilant monitoring of creatine kinase (CK) levels is essential for the timely detection of RML (56). Instances of RML serving as the initial indication for COVID-19 have been documented in pediatric patients, underscoring the importance of heightened clinical vigilance in any patient exhibiting indications or symptoms suggestive of RML (91). In severe cases, RML can precipitate complications, such as acute kidney injury, disturbances in electrolyte balance, and cardiac dysrhythmias. Notably, not all COVID-19 patients exhibiting RML manifest overt symptoms; some instances may only be discernible via laboratory assessments revealing heightened levels of muscle enzymes in the bloodstream.

### 5.3 Approach to RML secondary to COVID-19 infection

In COVID-19 patients, RML can be a complication that requires specific attention (9). Prompt recognition and management of RML in COVID-19 patients are essential to prevent further complications and optimize outcomes. Monitoring for symptoms of RML, such as muscle pain, weakness, and dark urine, is critical in COVID-19 patients to promptly address potential complications (56, 82). Patients diagnosed with RML require aggressive fluid therapy to mitigate the risk of acute kidney injury; however, it is essential to exercise caution in individuals with COVID-19 to prevent worsening of oxygenation and respiratory issues due to fluid overload. Prompt identification of this condition is imperative for effective management and prevention of potential complications (56). Treatment encompasses measures such as fluid administration, diuretic therapy, and, in severe instances, continuous renal replacement therapy (88). Timely identification and appropriate management are paramount in addressing COVID-19, underscoring the importance of precisely understanding its clinical manifestations (111).

## 6 Association between COVID-19 vaccination and rhabdomyolysis

Adverse effects associated with COVID-19 vaccination are generally mild and temporary, and serious complications are uncommon. Common side effects include discomfort, swelling, redness at the injection site, fatigue, headaches, muscle aches, chills, nausea, and fever. These effects typically subside within a few days and are comparable to those observed with routine vaccinations (112). However, there have been documented instances of RML occurring after the COVID-19 vaccination, albeit rarely. Cases have been reported in which individuals developed RML following the administration of COVID-19 mRNA vaccines, such as Comirnaty (BioNTech/Pfizer), have been reported. These cases underscore the importance of promptly identifying potential adverse reactions for appropriate management (113–115). The onset of RML after vaccination can vary, ranging from 5 to 10 days post-vaccination. Treatment typically involves intravenous fluids, with outcomes ranging from partial improvement to fatality (115). Although vaccination remains crucial for mitigating COVID-19 complications, healthcare providers should be vigilant about the rare occurrence of RML as a manageable adverse reaction to COVID-19 vaccination (116). Early recognition, diagnosis, and supportive care are crucial to ensure favorable outcomes for individuals who develop RML following vaccination (113–115).

### 6.1 Mechanism of RML secondary to COVID-19 vaccination

Although the mechanism underlying RML following COVID-19 vaccination has not been completely elucidated, it is proposed to be an uncommon nonspecific reaction to immunization rather than specific to mRNA or COVID-19 vaccines. Instances of RML have been reported as rare adverse events following mRNA COVID-19 vaccination, notably in patients receiving high-dose statins combined with fibrates (116). The muscle breakdown process after COVID-19 vaccination involves exposing muscles to modified contaminant agents through direct injection, provoking an immune response to the injected antigen. This immune response can induce muscle toxicity associated with the inciting agent, its constituents, and the host immune or inflammatory response. Although the precise mechanism causing damage to the injected muscle remains incompletely understood, toxic myopathy may contribute to reported pain at the vaccination site. In cases of RML post-COVID vaccination, skeletal muscle breakdown can occur following insult or injury, with potential triggers including trauma, excessive physical activity, immobilization, drug use, medications, or neuroleptic malignant syndrome (113, 116, 117). In the context of COVID-19 vaccination, instances of RML have been documented, with proposed mechanisms including autoimmune responses triggered by the vaccine and activation of CD4+ and CD8+ T cell responses against viral spike proteins (118). The proposed mechanisms for renal failure in RML include renal tubular obstruction due to myoglobin accumulation in the kidneys and free radical-mediated cytotoxicity, leading to tubular necrosis (119) (see Figure 1).

## 7 Conclusion

In conclusion, the increased incidence of RML in severe COVID-19 cases, likely driven by intense inflammation and potential direct viral invasion of muscle tissue, along with its rare occurrence following COVID-19 vaccination, underscores the need for heightened clinical vigilance. Early recognition and prompt management are essential to prevent serious complications, particularly through aggressive hydration and electrolyte correction. Clinicians should remain alert to the possibility of RML in patients presenting with neuromuscular symptoms post-vaccination and assess for cardiac involvement due to associated conditions like myocarditis. A thorough understanding of RML’s diverse presentations and risks in COVID-19 is critical for improving patient outcomes and enhancing safety during the pandemic.

It is important to acknowledge that the findings are primarily based on case reports, which may limit the generalizability of the conclusions drawn. Additionally, the lack of randomized controlled trials on RML associated with COVID-19 infection and vaccination presents a significant gap in the literature. Further research is necessary to investigate the processes contributing to mortality associated with RML and to develop therapies that could effectively improve patient outcomes within this group.
